# C-951-02. Dexamethasone and Its Effect on Postoperative Hyperglycemia in Burn Populations

**DOI:** 10.1093/jbcr/irag033.110

**Published:** 2026-04-06

**Authors:** Bassel Hafez, Samuel Boes, Samuel W Jones, Sadaf Akbari, Colette Galet, Alexander Kurjatko

**Affiliations:** University of Iowa Burn Center, Iowa City, IA; University of Iowa Carver College of Medicine, Iowa City, IA; University of Iowa Health Care, Iowa City, IA; University of Iowa Burn Center, Iowa City, IA; University of Iowa, Iowa City, IA; University of Iowa, Iowa City, IA

## Abstract

**Introduction:**

Postoperative nausea and vomiting (PONV) are common adverse events following anesthesia associated with patient morbidity. Dexamethasone (DXM) is a prophylaxis against PONV, with analgesic and anti-inflammatory benefits. However, its use is controversial in patients with diabetes mellitus due to its association with postoperative hyperglycemia. Burn injuries lead to metabolic derangements that result in augmented glucose levels. Herein, we investigated the relationship between DXM administration and the development of postoperative hyperglycemia in burn-injured patients.

**Methods:**

This was a retrospective cohort study. All adult burn patients admitted to our burn center from July 1, 2015 to June 30, 2024 who required at least one operation and had a postoperative serum glucose value recorded within 7 days of their first surgery during the index admission were included. Patients admitted for other traumatic injuries were excluded. Data were abstracted from our burn registry and electronic medical record. The primary exposure was perioperative DXM during the first burn-related surgery. Serum glucose levels were recorded pre- and post-operatively Primary outcomes were postoperative glucose levels and insulin needs postoperatively over 24 hours. Secondary outcomes were hospital length of stay and discharge disposition. All analyses were performed using SPSS 28.0 (IBM, Chicago, IL), and *p*<.05 was considered significant.

**Results:**

A total of 639 patients were included in this study; 278 received perioperative DXM. Patients in the DXM group were significantly younger (52 [33, 64] vs. 57 [40, 68] years, *p*=.004). Patients who received DXM were less likely to be male (66.5% vs. 77.3%, *p*=.003) and to present with inhalation injury (4.7%vs. 11.6%, *p*=.002). There were no differences in comorbidities or burn injury severity. Patients who received DXM had fewer ventilator days (7 [2 - 19] vs. 2 [1 - 4], *p*<.001), less burn-related surgeries (1 [1 - 2] vs. 2 [1 - 2], *p*<.001), and shorter hospital stays (12 [7-18] vs. 14 [9 - 23.5], *p*<.001). Postoperative hypotension (7.6% vs. 21.9%, *p*<.001) and mortality (0.4% vs. 4.7%, *p*=.001) were lower in the DXM group. On multivariate analysis, use of DXM was positively associated with hyperglycemia (Odd ratio = 1.77 [1.18 - 2.65], *p*=.006) and inversely associated with insulin requirement during the 24-to-48 hour period post-surgery (Odd ratio = 0.55 [0.36 - 0.85], *p*=.008).

**Conclusions:**

Perioperative DXM is a common medication given to burn patients for prophylaxis against PONV that is associated with postoperative hyperglycemia in burn-injured patients.

**Applicability of Research to Practice:**

Perioperative DXM is associated with postoperative hyperglycemia. Further study is necessary to determine the clinical implications of DXM use in burn patients.

**Funding for the study:**

N/A.

